# 

**Published:** 1871-08

**Authors:** 


SHOULD any of our subscribers or exchanges fail to get the COMPANION regularly, they will confer a favor by notifying us of the fact.

WE respectfully solicit Original Articles, Reports of Societies, Clinics, Home and Foreign Correspondence, News, etc., etc., of interest to medical readers.

To insure publication arictles should be practical, as brief as possible and carefully written, on one side of the paper. Articles should be received by the 15th of one month to insure publication in the next.

### TO OUR SUBSCRIBERS.

In this number we forward receipts of payments to, and bills of subscriptions for the COMPANION. We earnestly ask all our subscribers who have not paid, to make it a religious duty to do so at once. Two dollars is a small sum to any one subscriber, but put a number of these together, and they become of importance. If you have not yet paid do not fail to do so now. Let every subscriber do his duty, and he will receive the thanks of our very accommodating and obliging publisher. He can, in this way, discharge a small duty and receive the warmest of thanks for doing so. Friends, don ot disappoint our publisher, who, by his kindness, enables us to furnish the JOURNAL monthly to you. Two dollars is a very small sum, it is true, but should you forget to forward it you are sure to disappoint our publisher.

### OUR ASSOCIATED EDITORS.

With this issue of the COMPANION, we add to the number of our Associated Editors. These gentlemen are so well known as to make it unnessary for us to allude to them individually. Their contribu-

### BOOKS AND PAMPHLETS.

A Practical Treatise on the Diseases of Infancy and Childhood, by Thomas Hawkes Tanner, M. D., F. L. S., Author of the Practice of Medicine, Index of Diseases and their treatment, etc. Third American editioa, from the London edition and enlarged, by Alfred Meadows, M. D., London member of the Royal College of Physicians, etc., etc. Philadelphia, Lindsay & Blakison, 1871

We have received from the publishers, through Messrs. Lynch & Co., Atlanta, Ga., the above volume. Price $3 50. The value of the work before us cannot be too highly estimated, when it is remembered that the diseases of children constitute a large ele. ment in the practice of every physician, requiring careful study and the most industrious research in order to successfully meet them by appropriate treatment. The practitioner will be furnished very great aid by availing himself of this excellent treatise—perhaps the very best (as well as latest) on the subject.

The work is divided into four parts : Part I. Discusses the Physiology and Pathology of childhood, including chapters on the Anatomical and Physiological peculiarieties of childhood; on Hygiene, Pathology, Symptomatology and Therapeutics. Part II. Treats of general Diseases, including all the varieties of Fever—continued and eruptive—and the several Diathetic diseases—viz : Scrofula, Tuberculosis, Rickets and Syphilis. Part III. Embodies all the Special Diseases of children, arranged in their proper physiological order—viz : Diseases of the Nervous, Respiritory, Circulatory, Digestive and Urinary, Systems, with Diseases of the Skin, Ey eand Ear. Part IV. Discusses the more common Accidents, Injuries, Malformations and Deformities—congenetal and acquired—of childhood, including those connected with births. Lastly, a large and well arranged Appendix of Formulæ:

Special attention has been bestowed, by the Editor, upon diathisis, and we heartily concur with him in the opinion expressed that, " herein will be found the key to the successful treatment of most of the diseases of early life."

We cordially recommend the work to every diligent and consciencious physician.

Urethorcele, Catarah and Ulceration of the Bladder in Females, by Nathan Bozeman, M. D., New York. Wm. Baldwin & Co.,

177, Broadway, N. Y. We have read with much interest the pamphlet of Dr. Bozeman and recommend it to the attention of the profession.

Amputation of Redundant Scrotum in the treatment of varicocele, illustrated with a new instrument, by M. H. Henry, M. D., Surgeon to the New York Dispensatory, etc., etc. F. M. Christern, No. 77, University Place, New York. We have received the above pamphlet, "with the compliments of the author," and take pleasure in calling the attention of surgeons to it, as a valuable addition to the literature upon the subject.

Catarrh and Croupous Inflammation of Mucus Membranes, by Samuel G. Armor, M. D., Professor of the principles and practice of medicine and clinical medicine in the Long Island College Hospital, Brooklyn, N. Y.

This is a very valuable pamphlet. The author believes that we gain clear conceptions of certain morbid states, by the terms "catarrh" and "croupous," for the reason that they point out a definate pathological condition, peculiar to mucus membranes alone. Neimeyer defines catarrh as consisting in—Hyperæmia—abundant flow of mucus—increased detachment of epithelium. The author discusses the origin of the pus-cell in its relation to mucus membranes; and treats, consecutively, the catarrhal, suppurative true croupous, and diphtheritic inflammatory stages of the various mucus tracks of the body. He thinks these diseases should be studied with an interest and zeal pertaining to no other department of pathology.

The Half-Yearly Abstract of the Medical Sciences. Being a Digest of British and Continental Medicine, and of the Progress of Medicine and the Collateral Sciences. Edited by Wm. Domet Stone, M. D., F. R. C. S., vol. 53, July 1871. Philadelphia; Henry C. Lea.

This valuable Abstract has been regularly forwarded to us by the obliging publisher. It should be in the hands of every wide-awake practitioner. We know of no better investment—no finer intellectual medical feast for the money—than the one proposed by Henry C. Lea. For the small sum of six dollars he will forward to any address three magnificent periodicals—The Half-

Yearly Abstract—The American Journal of the Medical Sciences and The Medical News and Library, making in all about 1,500 large octavo pages per annum. The practitioner is the loser who does not fall in with this offer.

The Physiological Action and Theraputic use of Chloral, by J. B. Andrews, M. D., Assistant Physician New York State Lunatic Asylum (from the American Journal of Insanity).

We have received the above pamphlet, with the compliments of the author. Chloral, at one bound, has occupied a prominent position in the list of therapeutical agents. Every effort calculated to determine its true value is important to the Profession. For this purpose the pamphlet before us will serve a good end.

Historical Memoranda, relative to the discovery of Etherization, and the connection with it of the late Dr. Wm. T. G. Morton. Prepared by a committe of citizens of Boston, chosen to raise a Morton Testimonial Fund.

Color-Blindness, and its acquisition through the abuse of alcohol and tobacco, by Richard H. Derby, M. D., late Assistant Surgeon of Prof. Von Graffe, at Berlin. D. Appleton & Co., 540 and 551, Broadway, N. Y., 1871.

This pamphlet furnishes an additional argument against the abuse of alcohol and tobacco—articles which are abused, if used at all, except as medicines.

Artificial Induction of Labor in Uræmia. By Samuel C. Busey, M. D., Physician to the Louise Home, one of the physicians to the Childrens Hospital, D. C., and physician in charge of diseases of children at the Columbia Dispensary.

This is a very instructive and valuable monograph, of 62 pages, from the pen of one who is thoroughly conversant with his subject. Every practitioner would do well to read Dr. Busey's pamphlet.

A Review of Darwin's Theory of the Origin and Development of Man, by James B. Hunter, M. D., New York. D. Appleton & Co., 549 and 551, Broadway, 1871.

Who has not heard of Darwin's Theory? Yet, we opine, few have read his works. Should any of our readers wish to get the germ of his "speculations" and arguments advanced to sustain them, they will do well to secure the above pamphlet.

### COLLEGE ANNOUNCEMENTS.

We have received the following Annual College Announcements: Savannah Medical College, Session of 1871-72: For information, address W. Duncan, M. D., Dean of Faculty.

Annual Circular and Catalogue of the Bellevue Hospital Medical College, City of New York. Address Prof. Austin Flint, Jr., New York.

College of Physicians and Surgeons, New York. Medical Department of Columbia College. Sixty-fourth Annual Catalogue and Announcement. Address Jas. W. McLane, M. D., Secretary of the Faculty, 23d Street and 4th Avenue, New York.

Annual Circular of the Medical Department of the University of Louisianna. Session 1871–72. Address T. G. Richardson, M. D., Dean of Faculty.

Twenty-fifth Annual Announcement of the Medical Department of the University of Louisville, Ky. Address J. M. Bodine, Dean of Faculty.

Thirteenth Annual Announcement of the Chicago Medical Col. lege. Medical Department Northwestern University. The curriculum of this College are divided into three series or groups, corresponding with the three years of professional study. We trust all medical colleges will adopt this plan and rigidly enforce it. Address E. Andrews, M. D., Chicago, Ill.

University of the City of New York. Medical Department. Annual Announcement of Lectures. Address Prof. Henry Draper, M. D., 426, East 23d St., New York City.

Harvard University Medical School, 1871–72.

The plan of this school has been radically changed. Instruction will be uniformly distributed throughout the academic year. The course of instruction has been enlarged so as to extend over three years. This is a step in the right direction to elevate the standard of medical education. Address C. Ellis, M. D., 114 Boynton St., Boston, Mass.

Annual Announcement of the Medical Department of the University of Buffalo for the Session of 1871–72. Address J. F. Miner, M. D., 61 S. Deverin street, Buffalo, N. Y.

Catalogue and Announcement of the Univerity of Pennsylvania, for the One Hundred and Sixth Session, 1871–72, including the Auxiliary Faculty of Medicine and proceeding of the Society of the Alumni. For particulars, address R. E. Rogers, M. D., Dean of the Medical Faculty.

Third Annual Announcement of the Kansas City College of Physicians and Surgeons. Session of 1871–72. Address Dr. S. S. Todd, President, or Dr. J. L. Peed, Secretary of Faculty, Kansas City, Mo.

Other Announcements will be noticed when forwarded.

We have also received the following: The Brooklyn City Hospital Report, 1870, with trustees and officers for 1871.

The Medical and Scientific Circular and College Register, 1871. Wm. Baldwin & Co., 21 Park Row, New York. Price 25 cents.

Henry C. Lea's (late, Lea & Blanchard's) Classified Catalogue of Medical and Surgical Publications. An illustrated catalogue of 64 octavo pages, handsomely printed, will be forwarded by mail, post-paid, on receipt of ten cents. This is one of the largest and most enterprising medical publishing houses in the country. We will take great pleasure in noticing the publications of this house, when forwarded to us. Address Henry C. Lea, Nos. 706 and 708, Sansom St., Philadelphia, Pa.

Lee & Shepard's Monthly Bulletin of New Publications. Lee, Shepard & Dillingham, 47 and 40, Green St., New York. This will be forwarded, without charge, to all book-buyers. We will take pleasure in noticing any of their publications.

